# A restatement of the natural science evidence base relevant to the control of bovine tuberculosis in Great Britain^†^

**DOI:** 10.1098/rspb.2013.1634

**Published:** 2013-10-07

**Authors:** H. Charles J. Godfray, Christl A. Donnelly, Rowland R. Kao, David W. Macdonald, Robbie A. McDonald, Gillian Petrokofsky, James L. N. Wood, Rosie Woodroffe, Douglas B. Young, Angela R. McLean

**Affiliations:** 1 Department of Zoology, Oxford Martin School, University of Oxford, South Parks Road, Oxford OX1 3PS, UK; 2 WildCRU, The Recanati-Kaplan Centre, University of Oxford, South Parks Road, Oxford OX1 3PS, UK; 3 Department of Infectious Disease Epidemiology, MRC Centre for Outbreak Analysis and Modelling, Imperial College London, St Mary's Campus, Norfolk Place, London W2 1PG, UK; 4 Boyd Orr Centre for Population and Ecosystem Health, Institute of Biodiversity, Animal Health and Comparative Medicine, University of Glasgow, Bearsden Road, Glasgow G61 1QH, UK; 5 Environment and Sustainability Institute, University of Exeter, Cornwall Campus, Penryn, Cornwall TR10 9EZ, UK; 6 Disease Dynamics Unit, Department of Veterinary Medicine, University of Cambridge, Madingley Road, Cambridge CB3 0ES, UK; 7 Institute of Zoology, Zoological Society of London, Regent's Park, London NW1 4RY, UK; 8 MRC National Institute for Medical Research, The Ridgeway, Mill Hill, London NW7 1AA, UK

**Keywords:** bovine tuberculosis, epidemiology, cattle, badgers, vaccination

## Abstract

Bovine tuberculosis (bTB) is a very important disease of cattle in Great Britain, where it has been increasing in incidence and geographical distribution. In addition to cattle, it infects other species of domestic and wild animals, in particular the European badger (*Meles meles*). Policy to control bTB is vigorously debated and contentious because of its implications for the livestock industry and because some policy options involve culling badgers, the most important wildlife reservoir. This paper describes a project to provide a succinct summary of the natural science evidence base relevant to the control of bTB, couched in terms that are as policy-neutral as possible. Each evidence statement is placed into one of four categories describing the nature of the underlying information. The evidence summary forms the appendix to this paper and an annotated bibliography is provided in the electronic supplementary material.

## 1. Introduction

Bovine tuberculosis (bTB) is a major disease of cattle that can also affect humans, and many other livestock and wild animal species [1,2]. Human infection has not been a major public health problem in developed countries since the introduction of milk pasteurization [3]. Advanced cases in cattle experience loss of condition, and this directly affects the economic value of the animal, but in most developed countries detection of infection leads to movement restrictions being placed on the herd, mandatory slaughter and considerable indirect losses for the farmer [4].

The incidence and geographical distribution of bTB in Great Britain has been increasing for the last two decades [5] (see also appendix; box 1), and the English and Welsh governments estimate that they have spent £0.5 billion in the last decade on testing, compensation and research with further costs being borne by the agricultural industry. All cattle herds are tested regularly for bTB, more frequently in areas of high incidence. Confirmation of infection triggers restrictions on cattle sale and movement, and the withdrawal of ‘Official Tuberculosis Free Status’ [4]. To reduce the risks of infection, farmers are encouraged to adopt preventive biosecurity measures. Much attention has also been paid to reducing the risk of transmission from wildlife reservoirs, of which the most important in the British Isles is the European badger, *Meles meles* [1,2]. There are vaccines available for bTB that provide some protection to badgers and cattle, variants on those used to protect against human tuberculosis [6]. EU law currently prohibits the vaccination of cattle as it can mask the detection of infection. The vaccination of badgers is the subject of intense current research [6,7], and vaccination has been under way in Wales since 2012 [8].

Box 1. **Changes in incidence and distribution of bTB in Great Britian 1986–2012. (*a*) Changes in incidence, which varies seasonally. bTB testing was interrupted during the foot and mouth epidemic. (*b*) Increase in the geographical area affected by bTB, ‘hot’ colours indicating higher densities of farms where disease has been confirmed (official TB-free status withdrawal).**

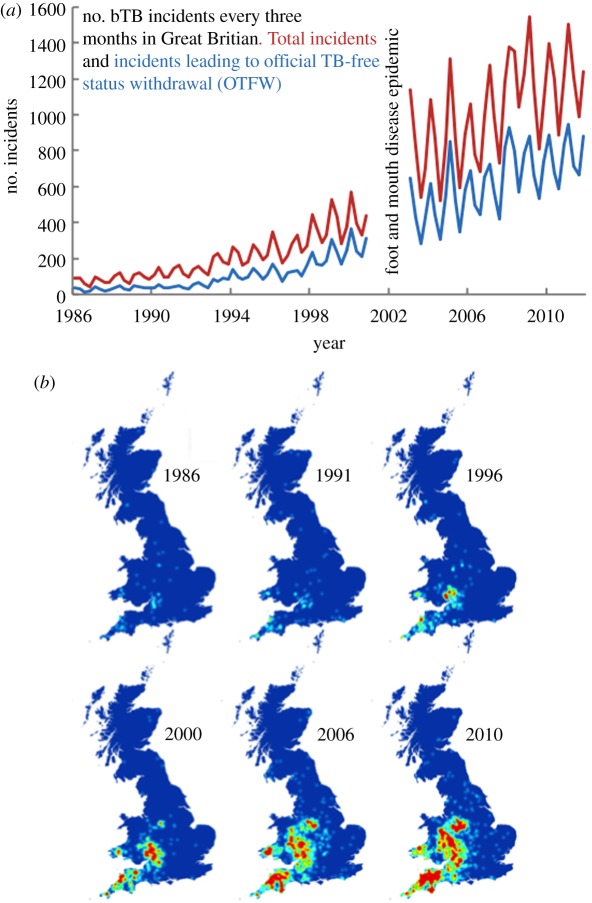

One strategy intended to reduce infection in wildlife reservoirs is culling. Badger culling was used routinely in the past [2], and its effectiveness was the subject of a major experiment, the Randomised Badger Culling Trial (RBCT), which ran from 1998 to 2006 [1,9]. Since then there has been no official badger culling, though the UK government has indicated its intention to allow culling in England, and badger culling at two pilot sites has been authorized for the summer of 2013 [10].

The prospect of badger culling has resulted in bTB policy becoming one of the most contentious areas of policy-making that involves science in the UK. The natural science evidence base is used by different sides to support different arguments, and exactly what constitutes natural science evidence has been called into question. The aim of the project described here is to provide a restatement of the relevant natural science evidence base written in a succinct manner comprehensible to non-expert readers and providing an entry into the technical literature. We have tried as far as possible to be policy-neutral, though realizing that this can never be absolute (the mere discussion of a strategy implicitly assumes it is a possible intervention). We hope that restating the scientific evidence will reveal the clear distinction between the science base, which is largely agreed, and the policy implications of that science base, which are hotly debated. This baseline summary also provides a natural starting point for a future review of evidence gaps.

## 2. Material and methods

The relevant literature on bTB in Great Britain was reviewed and a first draft evidence summary produced by a subset of the authors. At a workshop, most authors met to discuss the different evidence components and to assign to each a description of the nature of the evidence. Using existing systems such as GRADE [11], a tool for grading the quality of evidence used to support decisions in healthcare, we explored the restricted vocabulary used by the International Panel on Climate Change [12] to describe uncertainty associated with global environmental change, and ranking of evidence used by a study on bTB commissioned by the Welsh Government [13]. However, none precisely matched what we needed and instead we defined the following categories:

[D_ata_] A strong evidence base involving experimental studies or field data collection on bTB with appropriate detailed statistical or other quantitative analysis.
[E_xp_op_] A consensus of expert opinion extrapolating results from other disease systems and well-established epidemiological principles.
[S_upp_ev_] Some supporting evidence exists but further work would substantially improve the evidence base.
[P_rojns_] Projections based on available evidence for which substantial uncertainty exists that could affect outcomes.

These are explicitly not a ranking as, for example, some projections are firmly rooted in rich datasets, while some expert opinions are very much less so.

A revised evidence summary was produced and further debated electronically to produce a consensus draft. This was sent out to 25 scientists involved in bTB research, as well as to representatives from the farming industry, non-governmental organizations concerned with culling and Defra, the UK government department responsible for bTB policy. The document was revised in the light of much helpful feedback.

The project was funded by the Oxford Martin School (part of the University of Oxford), and though many groups were consulted, the project was conducted completely independently of any stakeholder.

## 3. Results

The summary of the natural science evidence base relevant to bTB policy-making in Great Britain is given in the appendix, with an annotated bibliography provided as the electronic supplementary material.

## 4. Discussion

We note several limitations of our project and how it might be extended.

First, the project considered only the natural science evidence base. There are very important social science issues involved with bTB policy-making that would also benefit from a formal evidence summary. For example, there are complex behavioural and behavioural economic aspects to the implementation of bTB control measures by the farming industry. Furthermore, the spectrum of possible interventions available to government is moulded by debate in civil society. The European Union's Common Agricultural Policy, and how each member state interprets it, shapes the economics of the livestock industry in Europe. The way agriculture is supported in England and Wales affects the structure of the countryside, including the wild animals that can harbour bTB. An entry into the social science literature on bTB is provided in the electronic supplementary material.

Second, the review concentrates on the evidence base from Great Britain. bTB is also a major problem in the Republic of Ireland, where badgers are a major reservoir. In Australia and New Zealand, successful efforts to control bTB have included targeting, respectively, introduced water buffalo (*Bubalus bubalis*) and brush-tailed possum (*Trichosurus vulpecula*), which act as reservoirs of infection. Differences in the regulatory and social structure of farming, the countryside, and the ecology of the different reservoirs all mean that lessons from other countries have to be taken with great caution, but the approach taken in this project might be usefully extended to consider more evidence from other countries. An entry into the literature on bTB control outside Great Britain is provided in the electronic supplementary material.

Finally, the review has largely concentrated on bTB epidemiology. We have not tried to summarize the evidence base relevant to the technical or operational logistics of culling or vaccination campaigns, nor the animal welfare consequences of different interventions.

We finish by stressing this is a consensus document written by the authors, and that we accept that a different group might have included or omitted different statements and might have categorized them in different ways. Policy-makers have to integrate evidence from the natural and social sciences, as well as to make political judgements about weighing the interests of different stakeholders. We hope the current summary will make it easier for evidence from the natural sciences to contribute to policy-making, and clarify where there is agreement and where dissent. We also hope that this restatement of the current evidence base will stimulate discussion about how to prioritize investment to address remaining uncertainties.

## Appendix A. A restatement of the natural science evidence base relevant to the control of bovine tuberculosis in Great Britain

For an annotated bibliography of the evidence supporting each statement, see the electronic supplementary material.

**(a) Introduction and aims**

(1) bTB is an infectious disease of cattle caused by the bacterium *Mycobacterium bovis*. In Great Britain, it can result in considerable economic losses to farmers, and costs to the taxpayer through testing and compensation for slaughtered animals. Control^1^ is difficult for several reasons, including the limited sensitivity of available diagnostic tests and because the pathogen also occurs in wildlife (especially badgers).
(2) The complex biology of the pathogen and its mode of transmission make the study of bTB epidemiology particularly challenging. Nevertheless, concerted research efforts over recent decades in Great Britain and elsewhere have generated a large body of important, policy-relevant information.
(3) The aim here is to provide a succinct summary of the evidence base relevant to policy-making for this disease in Great Britain as of June 2013. It also provides a consensus judgement on the nature of the different evidence components using the following descriptions with abbreviated codes.^2^
  [D_ata_] A strong evidence base involving experimental studies or field data collection on bTB with appropriate detailed statistical or other quantitative analysis.
  [E_xp_op_] A consensus of expert opinion extrapolating results from other disease systems and well-established epidemiological principles.
  [S_upp_ev_] Some supporting evidence exists but further work would substantially improve the evidence base.
  [P_rojns_] Projections based on the available evidence for which substantial uncertainty often exists that could affect outcomes.
(4) This document concentrates on the natural science evidence base; evidence from social sciences and economic analysis is also of great importance for policy-makers but is not included here. The document also largely concentrates on the evidence base from Great Britain. There is a need for a careful review of how lessons from bTB control in other countries with different farming systems or wildlife reservoirs can inform policy in Great Britain.
(5) Despite the substantial progress that has been made in understanding bTB the natural science evidence base cannot alone determine policy to control or eradicate the disease. All policy options have costs, benefits and risks that affect the stakeholders involved in different ways. Policy-makers inevitably have to consider and weight the interests of these stakeholders, as well as balancing uncertain benefits and potential risks in deciding what actions to take. Different weightings and balances can lead to different decisions. Nonetheless, it is critically important that all policy be informed by the evidence base and that policy-makers clearly distinguish the scientific and other (economic, social, ethical and political) inputs into the decisions that have to be made.

**(b) Epidemiology**

(6) The risk of bTB varies geographically within Great Britain; some areas have a consistently high incidence in cattle while infection has remained low or practically absent elsewhere. Annual herd testing for bTB is currently (2013) carried out over a large area of England (60 000 km^2^) and the entirety of Wales, though disease incidence varies within this region (box 1) [D_ata_].
(7) Since the mid-1980s, the incidence and geographical distribution of bTB in cattle has increased markedly in England and Wales (box 1) [D_ata_].
(8) Efforts to control the disease in cattle include regular testing of herds, destruction of individuals that test positive (37 068 cattle in 2012; a further 943 close contacts were also slaughtered) and post-mortem surveillance of all routinely slaughtered animals. Where infection is detected in a herd, cattle sale and movements are restricted and contacts of the infected herd traced [D_ata_].
(9) In recent decades, the observed pattern of bTB breakdowns^3^ in areas of low incidence has been correlated with cattle movements, mainly from high-incidence areas [D_ata_]. However, the causes for the gradual spread of high-incidence areas are not understood [E_xp_op_]. While herd breakdowns occur throughout Great Britain, areas of high incidence are observed in some regions (many parts of Wales, the Midlands and the West Country) but not in others (east and north England, Scotland) [D_ata_].
(10) In the UK, Republic of Ireland, New Zealand and parts of the USA, regions where the problem of bTB has not been eradicated by test and slaughter of cattle, there is persistent infection in wildlife [D_ata_].
  (a) A recent rapid decline in bTB in cattle in New Zealand has, in part, been associated with control of a wildlife host species (the introduced brush-tailed possum) [D_ata_]. The relevance of this programme to the British Isles is limited by the very different biology of the wildlife hosts involved and also because the rules governing cattle movement, disease compensation and other aspects of bTB policy are different in New Zealand [E_xp_op_].
(11) In England and Wales, farms that have had a herd breakdown suffer a recurrence more often than would be expected by chance, while many farms in high-incidence areas escape infection much more often than would be expected by chance; recurrence is a relatively rare event in low-incidence regions [D_ata_].
(12) Infection can persist in cattle in herds that have been tested clear of infection because of limited sensitivity of current tests [D_ata_] (and see paragraph 21). Cattle moved from breakdown herds (or herds that, because they are at higher risk of being infected, are subject to frequent testing) are more likely to seed new breakdowns than those from other classes of herd [D_ata_].
  (a) Transmission occurs within cattle herds, and movement of undetected infected cattle can lead to transmission between herds (see also paragraph 27). In ‘Officially Tuberculosis Free’ regions of Great Britain, such as Scotland, nearly all herd breakdowns can be convincingly attributed to cattle movement [D_ata_].
(13) In England and Wales, cases of bTB in cattle occur more frequently in regions that support higher densities of both badgers and cattle [S_upp_ev_]. At the more local level, most studies that have looked for an association between high badger densities and elevated cattle TB incidence have not found one [D_ata_].
  (a) Badgers thrive in mixed pasture and woodland landscapes [D_ata_], which is also where much cattle farming occurs; the national level correlation is partly but not wholly explained by habitat [S_upp_ev_].
(14) *Mycobacterium bovis* is transmitted within and between populations of badgers and cattle [D_ata_].
  (a) Similar genetic types (genotypes) of *M. bovis* are found, more often than would be expected by chance, in local cattle and badger populations [D_ata_]. Transmission from badgers to cattle is an important cause of herd breakdowns in high-incidence areas. In the RBCT proactive cull areas (see paragraph 30), it has been estimated that 50% of confirmed herd breakdowns in the year before culling began were because of badgers, though this figure has very broad confidence limits [D_ata_].
  (b) *Mycobacterium bovis* can be transmitted to badger populations from infected cattle [D_ata_].
  (c) Transmission occurs within wild badger populations [D_ata_]; there is insufficient evidence currently available to say definitively whether the disease can persist in British badger populations without on-going transmission from cattle [E_xp_op_].
  (d) Estimates of the prevalence of *M. bovis* infection within wild badger populations are difficult, with most tests having limited sensitivity. Estimates from the initial proactive culling area in the RBCT (where bTB incidence is high), based on post-mortem examination and culture, ranged from 2 to 38% (mean 14% from 8052 badgers [D_ata_], though these tests may fail to detect up to half of all infections). Of infected animals, 41% had visible lesions. Post-mortem surveys of badgers killed by road traffic (which might be a biased sample if infected individuals are more at risk) in the 1970s to 1990s gave rates of infection prevalence levels of up to 25% in cattle high-incidence areas [D_ata_].
(15) Little is known about how *M. bovis* is transmitted between badgers and cattle. Transmission may be indirect; for example, through contamination of pasture, feed and drinking water. Alternatively, direct transmission via aerosol droplets at close contact may occur, possibly inside farm buildings as well as outdoors. No quantitative estimates of any of these transmission rates or their relative importance are currently available [S_upp_ev_].
(16) *Mycobacterium bovis* can infect a range of wild mammals in Great Britain in addition to badgers [D_ata_]. In most situations, when compared with badgers, other wild species appear to constitute a low overall component of the risk of onwards transmission to cattle, though wild deer may be a potential, but probably localized, source of infection to cattle [S_upp_ev_].
(17) *Mycobacterium bovis* also infects farmed and park deer, goats, pigs, sheep and camelids (e.g. alpacas and llamas), as well as companion mammals [D_ata_]. These species probably do not constitute a major risk to cattle, because they have little contact with cattle, are relatively rare or are unlikely to transmit infection onwards. However, occasional transmission of *M. bovis* may occur from these hosts to cattle, to wildlife or directly to humans [S_upp_ev_].
(18) The basic reproduction number (*R*_0_) is defined as the number of secondary cases of a disease resulting from a primary case in a fully susceptible population. The only current estimates for between-herd *R*_0_ for bTB in Great Britain have been derived from a strategic model of the interaction between *M. bovis*, cattle and badgers, and lie in the interval 1.02–1.11 [P_rojns_].
(19) In designing bTB control programmes for known and potential high-incidence areas, benefits will be obtained from implementing effective measures that target the disease in both cattle and wildlife in the same area [E_xp_op_].
(20) The best type or combination of interventions may differ between high- and low-incidence areas [E_xp_op_].

**(c) Testing and surveillance**

(21) There are several different methods available for diagnosing bTB infection in cattle, either alive or at slaughter. None of these is 100% sensitive, which means that infected animals are sometimes missed (false negatives). The tests are also not 100% specific, which means that uninfected animals may sometimes be incorrectly identified as infected (false positives). Sensitivity and specificity are usually defined with reference to a gold-standard test, which acts as the definitive arbiter of whether an individual is infected. There is no such gold-standard for bTB, and post-mortem investigations, which probably miss some infections, have to be used instead. Furthermore, all tests detect the results of processes that develop over time during an infection so cannot detect the very first stages of infection; some cannot detect very long-established infections either. Finally, both sensitivity and specificity are concepts that apply to a test of a single animal, or of a whole herd, and the same test will have different sensitivity and specificity at the individual level and at the herd level. Taken together, these factors mean that even carefully executed estimates of sensitivity and specificity vary widely [D_ata_].
(22) The relationship between diagnostic status and infectiousness is not known in detail [E_xp_op_]. However, it is thought that animals that have developed antibody immune responses and animals with large numbers of lesions at post-mortem are (or were) more infectious [E_xp_op_].
(23) The single intradermal comparative cervical tuberculin test (SICCT or ‘skin’ test) is the approved stand-alone test for bTB infection in living cattle used in the UK and the Republic of Ireland. It has high specificity in individual animals, and a recent meta-analysis of current diagnostic tests found a median value for the animal-level specificity of the SICCT test to be above 99%. The same meta-analysis estimated the mean herd-level sensitivity to be 49% (95% credible interval 27–74%) [D_ata_].
  (a) Sensitivity can be increased by using a lower threshold to define infection, but at some cost to specificity [D_ata_].
  (b) Some genetic lines of cattle may have a predisposition to test negative with the SICCT test after having been exposed to infection [D_ata_]. It is not yet clear whether this reflects a reduced chance of becoming infected or failure to make immune responses that the test can detect. There is either a very different sensitivity or different rate of progression in young animals [D_ata_]. Other factors, such as infection with liver flukes, Johne's disease and parturition, can all reduce SICCT test sensitivity [D_ata_].
  (c) The SICCT test requires two farm visits to inject the tuberculin and then to assess the skin response. Because it relies on a somewhat subjective interpretation of the relative size of two swellings generated by an immunological response in the skin, there may be considerable variability in the interpretation of this test in the field [S_upp_ev_].
(24) The gamma interferon (IFNg) test is used as an auxiliary test to the SICCT test and has lower relative specificity (median animal-level specificity of 98% (95% credible interval 96–99%) in the meta-analysis cited above). As implemented, IFNg identifies some exposed cattle not identified by the skin test and has a median estimated animal-level sensitivity of 67% (95% credible interval 49–82%) [D_ata_].
  (a) The IFNg test requires only a single farm visit and is then conducted in the laboratory, where it can be more consistently interpreted [D_ata_].
  (b) As with the SICCT test, the sensitivity of the IFNg test is compromised in cattle co-infected with liver flukes or Johne's disease [D_ata_].
(25) Slaughterhouse testing provides important surveillance information in all regions [D_ata_]. In 2012, it accounted for nearly one-quarter of all new confirmed breakdowns in cattle herds across Great Britain [D_ata_].
(26) Tests to diagnose *M. bovis* infection in live badgers are available but are not currently suitable for use in a disease-control setting [D_ata_].
  (a) Testing requires capturing badgers to collect blood samples. There is a test (Brock TB StatPak) that can be used immediately at the capture site, but its sensitivity is poor (posterior median 50.4%, posterior probability interval 44.9–56.1%). IFNg is a more sensitive alternative (posterior median 79.9%, posterior probability interval 68.8–89.5%) but requires specialist facilities and takes longer to perform [D_ata_].

**(d) Biosecurity**

(27) Cattle movements, especially movements from high-incidence areas, are associated with increased risk of the onward transmission of bTB [D_ata_].
  (a) Pre-movement testing (which in England and Wales includes animals moving in or from high-risk areas) and to a lesser extent post-movement testing reduces the risk of onward transmission [D_ata_].
  (b) The standard interpretation of the SICCT test is used for pre-movement testing; this provides high specificity at the individual animal level but with a concomitantly limited sensitivity [D_ata_].
  (c) Some short-distance cattle movements and other interactions between cattle on nearby or linked premises will be unrecorded [D_ata_] and could result in cattle-to-cattle transmission of bTB, though the extent of this is not well quantified [E_xp_op_].
  (d) The requirement for post-movement testing in Scotland has been shown to provide an incentive to farmers to purchase cattle from low-disease areas [D_ata_] and so probably reduces the risk to the individual herds owned by these farmers, as well as limiting onward transmission [E_xp_op_].
(28) There are farm management strategies that could potentially reduce cattle-to-cattle transmission (for example, strict isolation of reactors and double-fencing to keep herds separate), though a strong evidence base to evaluate different strategies is currently lacking [E_xp_op_].
(29) There are many forms of farm management that could potentially interrupt the different transmission pathways between badgers and cattle. For example, excluding cattle from badger setts and latrines, and restricting badger access to feed stores, cattle barns and drinking troughs have been suggested as means to reduce the risk of transmission. However, the relative importance of the various routes is poorly known (see paragraph 15) and a strong evidence base to evaluate different strategies is currently lacking [E_xp_op_].

**(e) Culling badgers**

(30) Culling badgers can affect the incidence of confirmed bTB in cattle herds in Great Britain [D_ata_]. The most important evidence for this comes from a major study, the RBCT, which compared the effects of proactive, reactive^4^ and no culling conducted at 10 triplets of sites during 1998–2005.
  (a) The RBCT found that annual proactive culling, as conducted in the trial, resulted in a relative reduction in new confirmed cattle herd breakdowns inside culling areas, which persisted after the final culls in 2005 but subsequently diminished over a 6-year period (box 2) [D_ata_].
    BOX 2.
    Key results of the RBCT proactive culling.
    (i) The black lines show the percentage difference (with 95% confidence limits) in new confirmed herd breakdowns between sites subjected to proactive culling compared with no-cull areas. The red lines show the same information for land up to 2 km outside the proactive culling area compared with land up to 2 km outside the no-cull trial areas.
    (ii) Estimates at particular time points can be read from the graph. There are various ways to summarize these data; averages and confidence intervals for three time periods are as follows:
      **Table:**
      	average % change			
      	proactive culling area		areas surrounding cull	
      time period	central estimate (%)	95% confidence interval	central estimate (%)	95% confidence interval
      during trial	−23	−12 to −33	+25	−1 to +56
      after trial	−28	−15 to −39	−4	−26 to +24
      entire period	−26	−19 to −32	+8	−14 to +35
      The averages involving the post-trial period include 5 years of data; choosing a different time span would affect their values.
    (iii) The figures above are a comparison of cull and non-cull sites, and hence represent relative differences. As background incidence was rising throughout the monitoring period, absolute reductions in rates of new confirmed cattle herd breakdowns (compared with historical rates) would be smaller than the relative reductions shown here, and absolute increases would be larger than the relative increases shown here.
    (iv) RBCT culling had no impact on approximately 30% of cattle herd breakdowns, which are unconfirmed.
  (b) While proactive culling was being carried out, there was an increase in the incidence of confirmed herd breakdowns on land surrounding (within 2 km) the RBCT proactive culling areas, though this rapidly waned after culling stopped (box 2) [D_ata_].
  (c) Reactive culling was discontinued in 2003 because confirmed herd breakdowns in these areas were significantly higher^5^ than in no-cull areas [D_ata_]. Although the early suspension of reactive culling prompted debate over the causal interpretation of these primary results, subsequent analysis of data from within the reactive culling areas found that the presence and extent of badger-culling activity were associated with significantly increased risk of a confirmed herd breakdown on nearby farms, and that when compared with no-cull areas the breakdowns were more prolonged [D_ata_].
  (d) Culling in the RBCT had no effect (positive or negative) on the incidence of unconfirmed breakdowns [D_ata_].
(31) Culling badgers is known to disrupt badger social structure, and this has been shown to cause badgers to move more frequently and over longer distances [D_ata_]. This effect is known as perturbation. The idea that perturbation may result in increased disease transmission (to other badgers and to cattle) has been termed the ‘perturbation hypothesis’ or a ‘perturbation effect’ [E_xp_op_].
  (a) In the RBCT, culling consistently increased the prevalence of *M. bovis* infection in badgers [D_ata_], and this is likely to be explained by the perturbation hypothesis [E_xp_op_]. It is not known how the prevalence of infection in badgers changed after culling ended.
  (b) Increases in the prevalence of infection in badgers were especially marked in those proactive RBCT culling areas surrounded by weaker barriers to badger movement, on land close to culling area boundaries, and following proactive culls which were not conducted simultaneously across the entire area [D_ata_]. These findings are again consistent with the perturbation hypothesis [E_xp_op_].
  (c) Increased transmission from badgers to cattle because of a perturbation effect has been suggested as an explanation for the observed increase in herd breakdowns on the land surrounding the RBCT proactive culling areas, and also the observed increase in herd breakdowns in the RBCT reactive culling areas [P_rojns_].
(32) The RBCT can be used with care to help project the results of possible badger-culling strategies.
  (a) Factors likely to have contributed to the reductions in cattle bTB achieved by RBCT proactive culling include: the marked (approx. 70%) reduction in badger density; the use of geographical barriers to badger immigration where available; culls conducted simultaneously across entire areas and repeated annually over at least 4 years; access to a high proportion (approx. 70%) of land; and targeting of badgers on inaccessible land [S_upp_ev_]. Failures to implement such measures in any proposed cull are likely to reduce the magnitude of any beneficial effect or even cause detrimental effects [E_xp_op_].
  (b) Culling over larger geographical areas would be expected to move the balance of effects towards a net benefit. An extrapolation assuming culling as carried out in the RBCT suggests a roughly circular area of at least 150 km^2^ would be required to be confident of avoiding a net detrimental effect [P_rojns_].
  (c) An analysis assuming a circular 150 km^2^ area and proactive culling similar to that carried out in the RBCT predicted that over a 9.5 year period with proactive culling in the first 5 years there would be a relative reduction in confirmed herd breakdowns of 20–34% (central figure 27%) within the culled area. When the additional herd breakdowns in a peripheral 2 km-wide area are included, the overall impact falls to 3–22% (central figure 12%) or 8–24% (central figure 16%), depending on assumptions.^6^ These figures (including the widely quoted figure of 16%) should be treated as indicative and the actual result might differ markedly in either direction [P_rojns_].
  (d) It is not currently known whether alternative culling methods (e.g. shooting of free-ranging badgers or snaring) could reduce badger densities more or less effectively in Great Britain than the cage trapping used in the RBCT, nor how different reductions in badger numbers inside culling areas would influence impacts on cattle bTB on adjoining land [E_xp_op_]. In general, the more a proposed culling programme differs from the conditions tested in the RBCT, the more the results are likely to differ, either positively or negatively [E_xp_op_].
  (e) In order to have a major impact on national disease incidence, any culling would need to be conducted over very large geographical areas. Culling at this scale would have a marked impact on the national badger population but would be unlikely to cause regional extinctions [E_xp_op_].
  (f) Evidence suggests that small-scale or short-term badger culling (including reactive culling) may exacerbate the disease problem through a perturbation effect [S_upp_ev_]. Unlicensed (illegal) shooting is likely to have a similar effect [E_xp_op_].
(33) Estimates of badger density may be required to inform culling efforts; estimation can be done by various means, though all have a high level of imprecision, and the more accurate the method the more expensive and difficult it is to carry out^7^ [D_ata_].
(34) Earlier studies at single sites in Great Britain suggested that culling badgers reduced local bTB incidence in cattle, although inference is limited because of lack of statistical replication [S_upp_ev_]. There have also been important experimental studies in the Republic of Ireland that are not reviewed here [D_ata_] (and see paragraph 4).

**(f) Vaccination**

(35) To vaccinate cattle against bTB, a BCG vaccine (a live attenuated strain of *M. bovis* that is widely used in humans) exists, but it is not yet licensed for use in cattle and such use is currently prohibited by EU regulations.
  (a) The main protective effect for cattle vaccinated with BCG is to reduce the severity of disease. This is measured experimentally at post-mortem by comparing the extent of infection within the bodies of vaccinated and control cattle. A recent field trial in Ethiopia found that the carcasses of 13 vaccinated calves had 56–68% less disease than was seen in 14 control calves, the degree of protection varying according to the method used to measure disease burden within the carcass [D_ata_].
  (b) Neonatal cattle vaccination provides the best protection, though this wanes after 1 year, suggesting a role for re-vaccination [D_ata_].
  (c) BCG vaccination of cattle leads to false positive reactions using standard SICCT and standard IFNg tests for bTB [D_ata_].
  (d) Novel tests that allow differentiation of infected and vaccinated animals (DIVA) perform well on cattle in a research setting (95% relative sensitivity, 96% specificity) but have yet to be assessed in field trials [D_ata_].
  (e) Vaccination is likely to have little effect (positive or negative) on the course of existing infections in cattle [E_xp_op_].
  (f) If vaccinated cattle do become infected, it is likely that a reduction in the extent of disease will limit their infectiousness, reducing onward transmission to cattle and to wildlife [P_rojns_].
(36) An injectable BCG vaccine for badgers is licensed and is in use in the field. Major demonstration projects (and many smaller-scale projects) involving vaccination of badgers are taking place in Wales and England.
  (a) As with cattle, the main protective effect for vaccinated badgers is to reduce the severity and progression of disease upon challenge with *M. bovis* [D_ata_].
  (b) In a clinical field trial, BCG reduced the risk of vaccinated badgers testing positive to a test of progressed infection (i.e. seroconverting) by 74%, and reduced the risk of testing positive to any of the available live tests of infection by 54% [D_ata_].
  (c) In the same clinical field trial, BCG reduced the risk of infection of unvaccinated cubs in a vaccinated social group (probably because of the reduction in the infectiousness of vaccinated badgers). When more than a third of the social group was vaccinated, the risk to unvaccinated cubs was reduced by 79% [D_ata_].
  (d) Vaccination of badgers is likely to have little effect (positive or negative) on the course of existing infections in badgers [E_xp_op_].
  (e) Vaccination delivered by injection does not cause the badger to excrete BCG bacilli, nor has vaccination of previously infected badgers been seen to enhance the excretion of *M. bovis* [D_ata_].
  (f) Vaccination would be expected to reduce the prevalence of *M. bovis* infection within badger populations over time [E_xp_op_]. Its administration by trapping and injecting does not lead to a perturbation effect [D_ata_]. It would probably require annual administration because of cub births introducing new susceptible animals to the badger population [E_xp_op_].
  (g) While it is reasonable to expect vaccination of badgers to reduce the incidence of bTB in cattle in high-incidence areas, no trial has been conducted to assess the magnitude and timing of these effects [E_xp_op_].
(37) Oral vaccines are in development for use in badgers.
  (a) In common with the injected vaccine, laboratory trials have shown that the oral vaccine reduces the severity of the disease in vaccinated badgers [D_ata_].
  (b) Orally vaccinated badgers can secrete small amounts of BCG in their faeces, but at well below the dose that would sensitize cattle [D_ata_].
  (c) Initial field trials of baits that do not contain vaccine show that they can be delivered to a high proportion of the badger population [D_ata_].
  (d) The main technical challenge in the development of an oral vaccine is ensuring that individual badgers receive a sufficient dose of live BCG to result in immunity [S_upp_ev_].
  (e) The risk of cattle consuming the oral vaccine within badger baits (which might cause them to respond to SICCT tests as though they were infected) can be reduced by strategies such as placing baits down badger sett entrances [S_upp_ev_].
